# Complete mitochondrial genome of the hybrid grouper *Epinephelus lanceolatus* (♀) × *E. moara* (♂)

**DOI:** 10.1080/23802359.2018.1507633

**Published:** 2018-09-10

**Authors:** Jong Yeon Park, Ji Hun Yoon, Choong Hwan Noh, Min Joo Kang, In-Chul Bang

**Affiliations:** a Department of Life Science and Biotechnology, Soonchunhyang University, Asan, Republic of Korea;; b Marine Bio Resources Research Center, Korea Institute of Ocean Science and Technology, Busan, Republic of Korea

**Keywords:** Hybrid grouper, *Epinephelus lanceolatus*, *E. moara*, complete, mitogenome

## Abstract

The complete mitochondrial genome of the novel hybrid grouper (*Epinephelus lanceolatus ♀* and *E*. *moara ♂*) includes 13 protein-coding genes, 2 ribosomal RNAs, 22 transfer RNA genes, and 1 control region (D-loop), for a total length of 16,743 bp. The overall nucleotide compositions encoded on the heavy strand are 29.73% A, 28.84% C, 15.08% G, and 26.64% T.

We report the complete mitogenome of a novel hybrid grouper between *Epinephelus lanceolatus* (♀) and *E*. *moara* (♂) in Tongyeong, Republic of Korea (34°49′36.8″N 128°20′01.6″E). Fry of the hybrid were sampled in 70% ethyl alcohol after immediately hatching in October, 2017. The extracted DNA specimen for hybrid grouper is stored at Soonchunhyang University in Republic of Korea (voucher number of SUC-14155). The complete mitochondrial genome (GenBank accession number MH123894) includes 13 protein-coding, 2 ribosomal RNA (rRNA), and 22 transfer RNA (tRNA) genes, and 1 control region (D-Loop), for a total length of 16,743 bp. The encoded genes are similar to those of other Serranidae (Kim et al. 2016; Tang et al. 2016; Wang et al. 2016). Most genes are encoded on the heavy strand, except for NADH dehydrogenase subunit 6 (ND6). Eight tRNA genes (*tRNA-Gln*, *Ala*, *Asn*, *Cys*, *Tyr*, *Ser*, *Glu*, and *Pro*) are encoded on the light strand. The total length of the 13 protein-coding genes (PCGs) is 11,429 bp. Eleven PCGs start with ATG; the two others are ATP6 (starts with CTG) and COX1 (starts with GTG). There are three types of stop codons: TAA at seven PCGs (ATP6, ATP8, COX1, ND1, ND4L, ND5, and ND6), incomplete T at five PCGs (COX2, CYTB, ND2, ND3, and ND4), and incomplete TA at COX3. The TAA stop codon of six PCGs (COX2, COX3, CYTB, ND2, ND3, and ND4) is completed by adding 3′ A residues to the mRNA. The overall nucleotide compositions encoded on the heavy strand are 29.73% A, 28.84% C, 15.08% G, and 26.64% T. The G + C content (43.92%) is less than that of A + T (56.37). The length of the mitochondrion DNA is 16,694 bp, which is 47 bp longer than that of *E*. *moara* (Xiao et al. 2016). The composition revealed differentiation with *E*. *moara* in the nucleotide frequency of 1.13% A, 0.02% C, 1.01% G, and 0.19% T. The 22 tRNA genes vary in length from 68 bp to 76 bp. The 12S rRNA, with a length of 953 bp, is located between tRNA-Phe and tRNA-Val, and the 16S rRNA, with a length of 1706 bp, is located between tRNA-Val and tRNA-Leu. The control region (D-loop), with a length of 1041 bp, is located between tRNA-Pro and tRNA-Phe. Molecular phylogenetic tree was constructed based on complete mitochondrion genes among 12 species of *Epinephelus*, 3 species of *Cephalophlis*, and 2 species of *Hyporthodus*. The phylogenetic tree was constructed using maximum likelihood (Figure 1).

**Figure 1.**
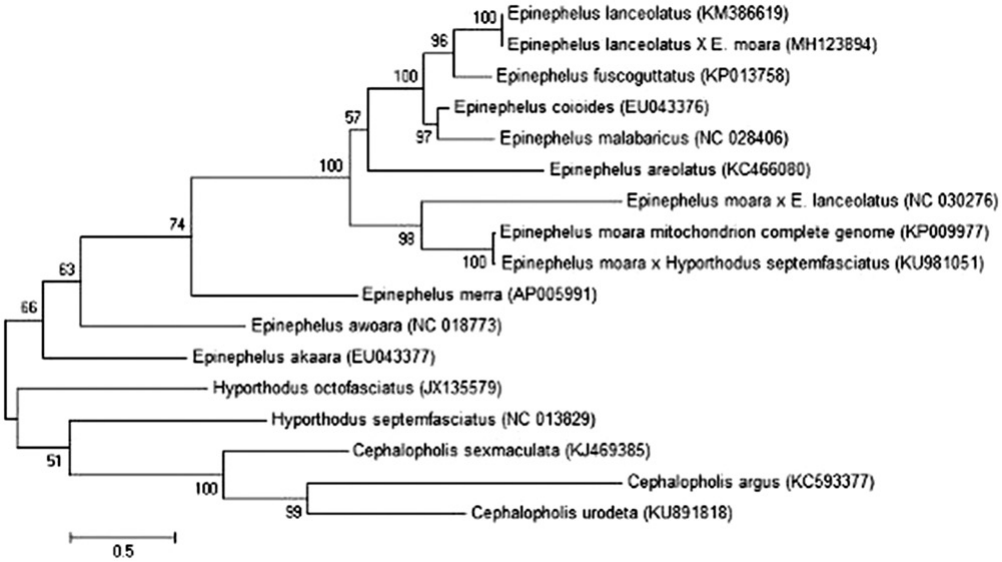
Molecular phylogenetic tree of complete mitochondrion genes among 12 species of *Epinephelus*, 3 species of *Cephalopholis*, and 2 species of *Hyporthodus*. The phylogenetic tree was constructed using maximum likelihood.
